# Coronarin D, a Metabolite from the Wild Turmeric, *Curcuma aromatica*, Promotes the Differentiation of Neural Stem Cells into Astrocytes

**DOI:** 10.1021/acs.jafc.2c00020

**Published:** 2022-03-04

**Authors:** Satoshi Otsuka, Midori Kawamura, Shutaro Fujino, Fumiaki Nakamura, Daisuke Arai, Nobuhiro Fusetani, Yoichi Nakao

**Affiliations:** † Department of Chemistry and Biochemistry, Graduate School of Advanced Science and Engineering, Waseda University, 3-4-1 Okubo, Shinjuku-ku, Tokyo 169-8555, Japan; ‡ Research Institute for Science and Engineering, Waseda University, 3-4-1 Okubo, Shinjuku-ku, Tokyo 169-8555, Japan; § Fisheries and Oceans Hakodate, 3-1-1 Minato-cho, Hakodate 041-8611, Japan

**Keywords:** neural stem cell, astrocyte differentiation, wild turmeric, coronarin D, labdane diterpenes

## Abstract

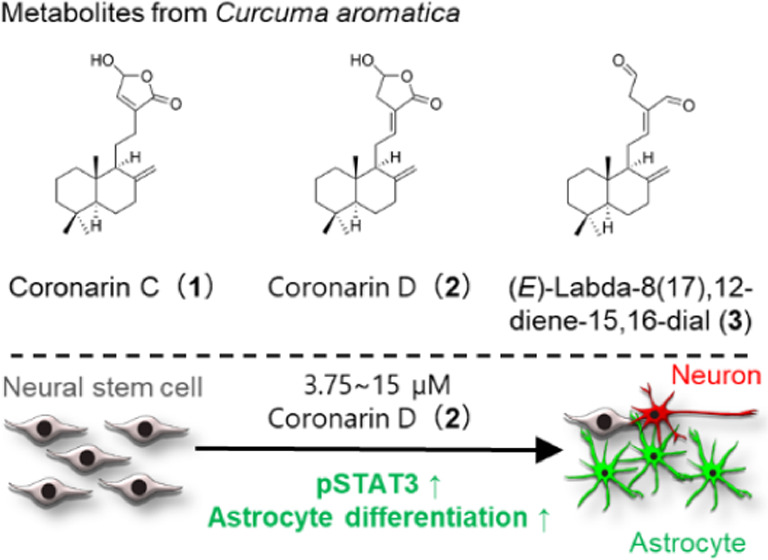

Plants in the genus *Curcuma* have been widely used as traditional medicines in Asian countries. These plants contain bioactive compounds with neuroprotective properties or activities that increase neural stem cells (NSCs) and neurons. However, bioactive components in *Curcuma* that promote the differentiation of NSCs into astrocytes have not yet been reported. Here, the effects of *Curcuma* extracts on the *in vitro* differentiation of embryonic stem-cell-derived NSCs were evaluated. The extract of the wild turmeric, *Curcuma aromatica*, strongly promoted the differentiation of NSCs into astrocytes. Bioassay-guided isolation yielded coronarins C (**1**) and D (**2**), as well as (*E*)-labda-8(17),12-diene-15,16-dial (**3**) as the bioactive compounds. Coronarin D (**2**) markedly promoted the differentiation of NSCs into astrocytes up to approximately 4 times (3.64 ± 0.48) and increased the expression level of GFAP at the mRNA and protein level, while compounds **1** and **3** exhibited only weak effects, suggesting that the 15-hydroxy-Δ^12^-γ-lactone moiety is important for bioactivity. Moreover, compound **2** increased the number of pSTAT3-positive cells, suggesting that compound **2** promoted astrocytic differentiation through JAK/STAT signaling pathway.

## Introduction

Plants in the genus *Curcuma* in the Zingiberaceae family have been widely used as traditional medicines in Asian countries, especially India and China.^1^ Many bioactive components have been isolated from *Curcuma* species, including *Curcuma longa* (turmeric) and *Curcuma aromatica* (wild turmeric).^2^ Curcumin,^3^ demethoxycurcumin, and bisdemethoxycurcumin are considered anti-inflammatory, neuroprotective, and antioxidant curcuminoids.^4^ In addition to curcuminoids, a wide variety of terpenoids with antibacterial, antitumor, or other pharmacological properties form another class of bioactive components from the genus *Curcuma*.^5−7^

Among many bioactivities of *Curcuma*, the neuroprotective property of *Curcuma* has attracted the attention of researchers, and curcumin has been shown to be neuroprotective through its antioxidative, anti-inflammatory, and anti-protein aggregating properties.^8^ Curcumin also inhibits neuroinflammation involved in the progression of neurodegenerative diseases by reducing the expression of inflammatory cytokines, including IL-1β, IL-6, and TNF-α.^9^

In addition to its neuroprotective activities, some *Curcuma* compounds were reported to affect the proliferation and differentiation of neural stem cells (NSCs). Curcumin stimulates the proliferation^10^ or differentiation of NSCs into neurons,^11^ and the aromatic compound turmerone, another major *Curcuma* component, was shown to increase the number of NSCs and promote neuronal differentiation.^12^

NSCs are distributed in brain regions such as the hippocampus and the lateral ventricles, and provide neurons and glial cells, such as astrocytes and oligodendrocytes, throughout the life span.^13−15^ Dysfunction of neural cells such as NSCs, neurons, and glial cells are deeply involved in neurodegenerative diseases such as Alzheimer’s disease,^16^ Parkinson’s syndrome,^17^ and depression.^18^ Therefore, the proliferation and differentiation of NSCs are potential targets for neuroprotective medicines and supplements.

Although numerous bioactive compounds promoting neuronal differentiation have been discovered in *Curcuma*, there are yet no reports of compounds promoting astrocytic differentiation. Bioassays using astrocytes or NSCs derived from pluripotent stem cells have been recognized as a new approach for studying neurogenesis or neurodegenerative diseases *in vitro* from the viewpoint of animal welfare.^19^ Some researchers have developed neural differentiation methods using NSCs derived from pluripotent stem cells^20,21^ and have used them to test neural toxicity or neuroprotective activity.^22,23^ Therefore, in this study, *Curcuma* components promoting astrocytic differentiation of NSC derived from mouse embryonic stem cells (ESCs) were searched for. The successful isolation, identification, and characterization of coronarin D and its analogues as bioactive substances in *C. aromatica* as well as their activities on astrocytic differentiation of NSCs are described.

## Materials and Methods

### Experimental Equipment for Structure Elucidation

All NMR spectra were acquired using Avance 400 or 600 MHz NMR spectrometer (Bruker Corporation, Billerica, MA). Liquid chromatography electrospray ionization tandem mass spectrometry (LC-ESI-MS) data were obtained using a Shimadzu UFLC XR liquid chromatography apparatus (Shimadzu Corporation, Kyoto, Japan) equipped with a TripleTOF 4600 system (AB Sciex LLC, Framingham, MA).

### Chemicals

MeOH (99.8%), MeCN (99.5%), CHCl_3_ (99.0%), and dimethylsulfoxide (DMSO) (99.0%) were purchased from Fujifilm Wako Pure Chemical Corporation (Osaka, Japan).

### Extraction and Isolation

The processed products of *C. aromatica* (tablets, 110 g; Nakazen Corporation, Okinawa, Japan) were first extracted with MeOH. This extract was subjected to octadecylsilyl (ODS) flash column chromatography (⌀2.0 cm × 3.0 cm) using a stepwise solvent system of MeOH/H_2_O (5:5 and 7:3), MeCN/H_2_O (7:3 and 85:15), MeOH, and CHCl_3_/MeOH/H_2_O (6:4:1). The bioactive fraction, fr.2-4 [eluted with MeCN/H_2_O (85:15)], was separated by silica gel open column chromatography (⌀3.0 cm × 10.0 cm) with the solvent system of CHCl_3_, CHCl_3_/MeOH (95:5 and 9:1), CHCl_3_/MeOH/H_2_O (8:2:0.1 and 7:3:0.5), and MeOH to yield 77 test tube fractions. The obtained 77 tubes were divided into 17 fractions (fr.3-1-17) monitored by thin-layer chromatography (TLC) pattern. The most active fraction, fr.3-7, was purified by reversed-phase HPLC [COSMOSIL 5C_18_-AR-II (Nacalai Tesque, Kyoto, Japan), with MeCN/H_2_O (7:3)] to give compounds **1** (fr.6-7) and **2** (fr.6-5) as the active substances.

The fresh rhizomes of *C. aromatica* (520 g wet weight) were extracted with MeOH. The extract was subjected to ODS flash chromatography [⌀5.0 cm × 10.0 cm, MeOH/H_2_O (5:5 and 7:3), MeCN/H_2_O (7:3 and 85:15), MeOH, CHCl_3_/MeOH/H_2_O (6:4:1)] to yield six fractions (fr.17-1-6). The fourth fraction, fr.17-4 [eluted with MeCN/H_2_O (85:15)] was fractionated by silica gel open column chromatography [⌀3.0 cm × 10.0 cm, CHCl_3_, CHCl_3_/MeOH (95:5 and 9:1), CHCl_3_/MeOH/H_2_O (8:2:0.1 and 7:3:0.5), MeOH] to yield 11 fractions (fr.18-1-11). One of the obtained fractions (fr.18-6) was purified by reversed-phase HPLC [COSMOSIL 5C_18_-AR-II, MeCN/H_2_O (7:3)] to give fr.19-2, containing **2**. The other fractions (fr.18-3) were also purified by reversed-phase HPLC [CAPCELL PAK C_18_ UG120, MeCN/H_2_O (75:25): Osaka Soda Corporation, Osaka, Japan] giving compound **3** as the other active substance. Purification schemes for compounds **1**–**3** are described in Figure S1.

### Cell Culture

The J1 mouse ESC line was purchased from the American Type Culture Collection (Manassas, VA) and maintained with mitomycin C (Fujifilm Wako Pure Chemical Corporation)-treated mouse embryonic fibroblasts (Kitayama Labes CO., LTD., Nagano, Japan) on 0.1% gelatin (Merck Millipore, Darmstadt, Germany)-coated dishes in Dulbecco’s modified Eagle’s medium (DMEM) (Fujifilm Wako Pure Chemical Corporation) containing 15% fetal bovine serum (FBS) (Biowest, Nuaillé, France), 1% l-glutamine (Thermo Fisher Scientific, Waltham, MA), 1% non-essential amino acids (Thermo Fisher Scientific), 1% penicillin/streptomycin (P/S) (Thermo Fisher Scientific), 0.18% 2-mercaptoethanol (Thermo Fisher Scientific), and 1000 U/mL LIF (Merck Millipore).

### *In Vitro* Differentiation of ESCs into NSCs

NSCs were induced from mouse ESCs using a previously reported method, with some modifications.^24,25^ Briefly, embryoid bodies (EBs) were formed with the hanging drop method using 7500 ESCs in 20 μL of medium in the absence of LIF for 3 days. The obtained EBs were transferred to a low-adhesion plate (Corning Inc., Corning, NY) and cultured in Neuron Culture Medium (Fujifilm Wako Pure Chemical Corporation) supplemented with 20 ng/mL rhEGF (R&D Systems, Minneapolis, MN) and 20 ng/mL rhFGF-2 (R&D Systems) for 96 h. Thereafter, the EBs were transferred to matrigel (BD Biosciences, Franklin Lakes, NJ)-coated dishes and incubated in NSC maintenance medium, MACS NeuroMedium (Miltenyi Biotec, Bergisch Gladbach, Germany) containing 2% MACS NeuroBrew-21 (Miltenyi Biotec), 1% P/S, 20 ng/mL rhEGF, and 20 ng/mL rhFGF-2, for 20 days. Finally, NSCs that migrated radially outward from the EBs were collected and cryopreserved until use in the *in vitro* NSC differentiation assay.

### *In Vitro* NSC Differentiation Assay

NSCs induced from ESCs were seeded into each well of matrigel-coated 96-well plates (Corning Inc.) at a density of 1 × 10^4^ cells/well and cultured for 72 h in the NSC maintenance medium. Then, the medium was replaced with fresh NSC maintenance medium or NSC differentiation medium, DMEM/Ham’s F-12 (1:1) (Fujifilm Wako Pure Chemical Corporation) containing 2% MACS NeuroBrew-21, 1% P/S, and 1% FBS, for 72 h. During this step, test samples dissolved in DMSO (Fujifilm Wako Pure Chemical Corporation) were added to the medium at a 1000-fold dilution. The differentiation rate of NSCs into astrocytes was calculated using the immunocytochemistry method described below. The bioactivity of the sample for NSC differentiation was evaluated by comparing the differentiation rate with that of the control (0.1% DMSO).

### Immunocytochemistry

Cells were washed with phosphate-buffered saline (PBS) (Takara Bio, Shiga, Japan) twice and incubated with 4% paraformaldehyde (Fujifilm Wako Pure Chemical Corporation) at 4 °C for 30 min. After PBS washing, the cells were incubated with PBS containing 5% skim milk (Fujifilm Wako Pure Chemical Corporation) and 0.2% Triton X-100 (Thermo Fisher Scientific) at 4 °C for 30 min. Then, the cells were treated with 5% skim milk solution containing primary antibody anti-GFAP (an astrocyte marker, mouse monoclonal antibody, 1:500, Merck Millipore) at 4 °C overnight. After washing with 5% skim solution, the cells were incubated with 5% skim milk solution containing secondary antibody Alexa Fluor 488-conjugated anti-mouse IgG (1:1000, Thermo Fisher Scientific) at room temperature for 2 h. After washing with 0.2% Triton X-100 solution, 0.2% Triton X-100 solution containing Hoechst 33342 (1:1000; Dojindo, Kumamoto, Japan) was added to visualize the nuclei, and the fluorescent cell images were obtained under the microscope (IX71, Olympus Corporation, Tokyo, Japan). The obtained images were analyzed by CellProfiler software,^26^ and the ratio of the number of GFAP-positive cells to that of the control was calculated as the rate of NSC differentiation into astrocytes.

### Real-Time Polymerase Chain Reaction (PCR)

Total RNA from cells was extracted using the RNeasy Plus Mini Kit (Qiagen, Hilden, Germany), and cDNA was synthesized using PrimeScript II 1st Standard cDNA Synthesize Kit (Takara Bio). Real-time PCR analyses were conducted on a 7500 Real Time PCR System (Applied Biosystems, Foster City, CA) using Thunderbird SYBR qPCR Mix (Toyobo, Osaka, Japan). The primer sequences used were as follows: 5′-TGGTGAAGGTCGGTGTGAAC-3′ and 5′-AATGAAGGGGTCGTTGATGG-3′ for *Gapdh* and 5′-CCATTCCTGTACAGACTTTCTCCAA-3′ and 5′-GGCCTTCTGACACGGATTTG-3′ for *Gfap*.

### Western Blotting

Whole proteins of cells were extracted with sample buffer solution (Nacalai Tesque, Kyoto, Japan), incubated at 95 °C for 5 min, and then centrifuged at 15,000 rpm for 5 min at 4 °C. Supernatants were subsequently separated by sodium dodecyl sulfate-polyacrylamide gel electrophoresis (SDS-PAGE) with a gradient gel (Atto, Tokyo, Japan), followed by electrophoretic transfer onto PVDF membrane (Merck Millipore). After the blotting, the membranes were blocked in Blocking One (Nacalai Tesque) for 45 min and then incubated with primary antibodies [anti-GFAP mouse monoclonal antibody (1:2500, Merck Millipore) and anti-ACTB mouse monoclonal antibody (1:2500, Santa Cruz, Dallas, TX)] at 4 °C overnight, followed by incubation at room temperature for 2 h with HRP-conjugated secondary antibodies (Santa Cruz). The Can Get Signal Immunoreaction Enhancer Solution Kit (Toyobo) was used as an antibody diluent for the signal enhancement. Signal was detected with LAS-4000 (GE Healthcare, Chicago, IL) using Chemi-Lumi One L (Nacalai Tesque), and signal intensities were calculated with ImageQuant TL Software (GE Healthcare).

### Flow Cytometry

Cells were fixed with 4% paraformaldehyde and blocked in PBS with 5% skim milk and 0.2% Triton X-100 at 4 °C for 30 min, respectively. Then, the cells were reacted at room temperature for 2 h with the antibodies of anti-GFAP mouse monoclonal antibody (1:1000, Merck Millipore) or anti-pSTAT3 rabbit monoclonal antibody (1:500, Cell Signaling Technology, Danvers, MA), followed by reaction at 4 °C for 30 min with Alexa Fluor 647-conjugated anti-mouse IgG (1:1000; Thermo Fisher Scientific) or Alexa Fluor 546-conjugated anti-rabbit IgG (1:1000; Thermo Fisher Scientific), respectively. The cells were then subjected to flow cytometry analysis with CytoFLEX S (Beckman Coulter, Brea, CA, Japan).

### Statistical Analysis

In Figure 4A,B, one-way ANOVA was used to evaluate statistical differences among each independent group, and Tukey’s multiple comparison test was used to assess differences between two groups using EZR,^27^ a graphical user interface for statistical analysis software R (R Foundation for Statistical Computing, Vienna, Austria). In Figures 4C–E and S15, Student’s *t*-test was used to test differences between two independent groups with Microsoft Excel (Seattle, WA).

## Results

### Evaluation of Extracts of the Wild Turmeric, *C. aromatica*, Using an *In Vitro* NSC Differentiation Assay

The processed products of the wild turmeric *C. aromatica* were first extracted with MeOH and subjected to ODS flash column chromatography [⌀2.0 cm × 3.0 cm, MeOH/H_2_O (5:5 and 7:3), MeCN/H_2_O (7:3 and 85:15), MeOH, CHCl_3_/MeOH/H_2_O (6:4:1)]. The obtained six fractions (fr.2-1-6) were evaporated and then diluted in DMSO to a concentration of 10 mg/mL.

**Figure 1:**
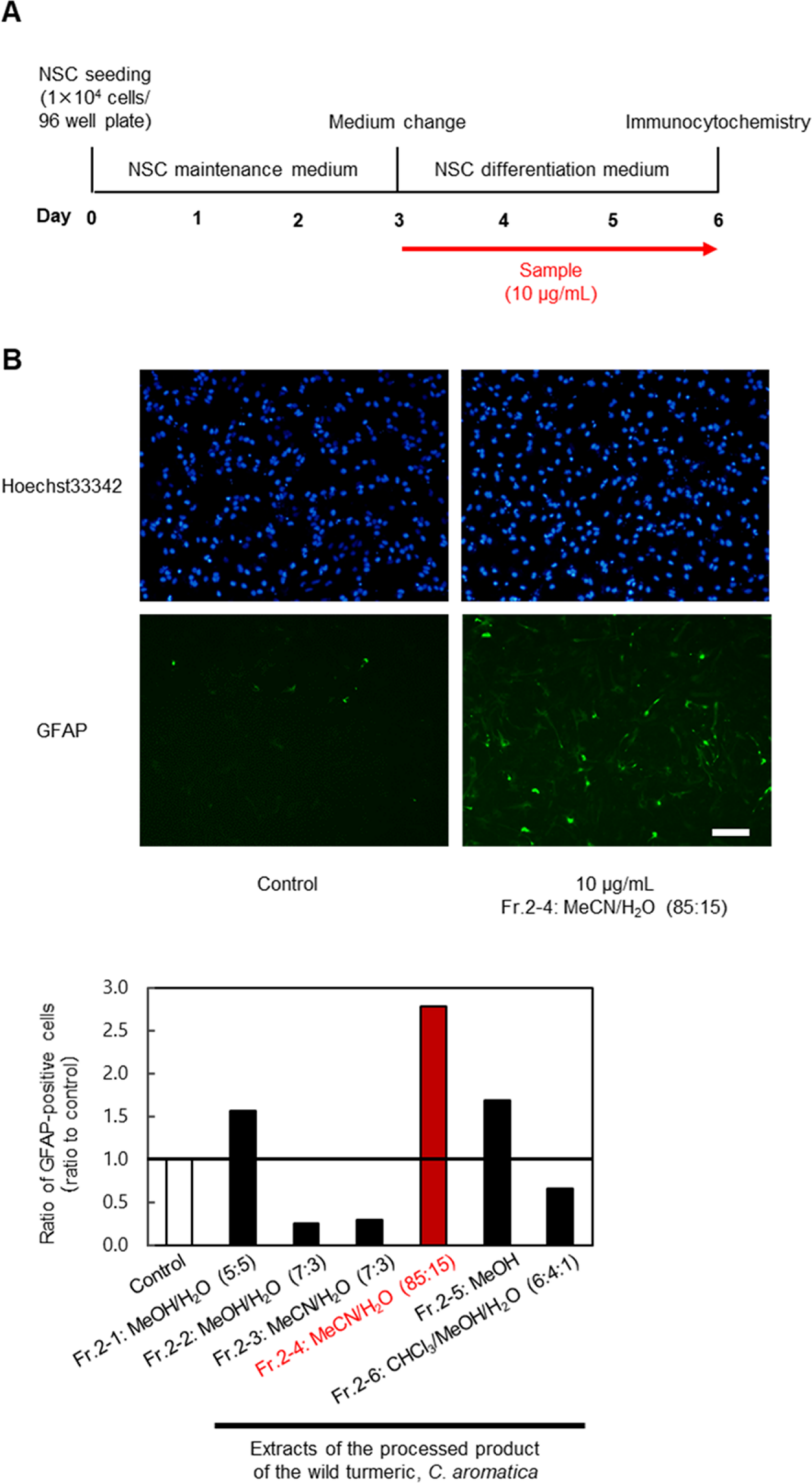
Evaluation of extracts of the wild turmeric *C. aromatica* using an *in vitro* NSC differentiation assay. (A) Scheme of the assay for the evaluation of effects by test samples on neural differentiation. (B) Result of the evaluation of fractions prepared from the processed product of the wild turmeric *C. aromatica*. NSCs were treated with DMSO or one of the *C. aromatica* fractions (10 μg/mL). Fluorescent microscopic images of astrocytes differentiated from NSCs treated with DMSO (left) or 10 μg/mL of fr.2-4 (right) are shown (blue: Hoechst 33342; green: Alexa Fluor 488-labeled GFAP; scale bar: 100 μm). Ratios of GFAP-positive cells were compared to the control (*n* = 2, mean).

The solutions of fr.2-1-6 were tested for bioactivity using an *in vitro* NSC differentiation assay (Figure 1A). Among the six fractions, fr.2-4, eluting with MeCN/H_2_O (85:15), markedly promoted astrocytic differentiation, with more than 2.5 times the number of GFAP-positive cells compared with control (Figure 1B).

### Purification and Identification of Bioactive Compounds from the Processed Product of the Wild Turmeric *C. aromatica*

The bioactive fraction, fr.2-4, was separated using silica gel open column chromatography [CHCl_3_, CHCl_3_/MeOH (95:5 and 9:1), CHCl_3_/MeOH/H_2_O (8:2:0.1 and 7:3:0.5), and MeOH] to yield 77 test tube fractions. The 77 fractions were divided into 17 fractions (fr.3-1-17) monitored by the TLC pattern. The most active fraction, fr.3-7, was further purified by reversed-phase HPLC, COSMOSIL 5C_18_-AR-II, with MeCN/H_2_O (7:3), giving compounds **1** (fr.6-7) and **2** (fr.6-5) as the active substances.

**Figure 2:**
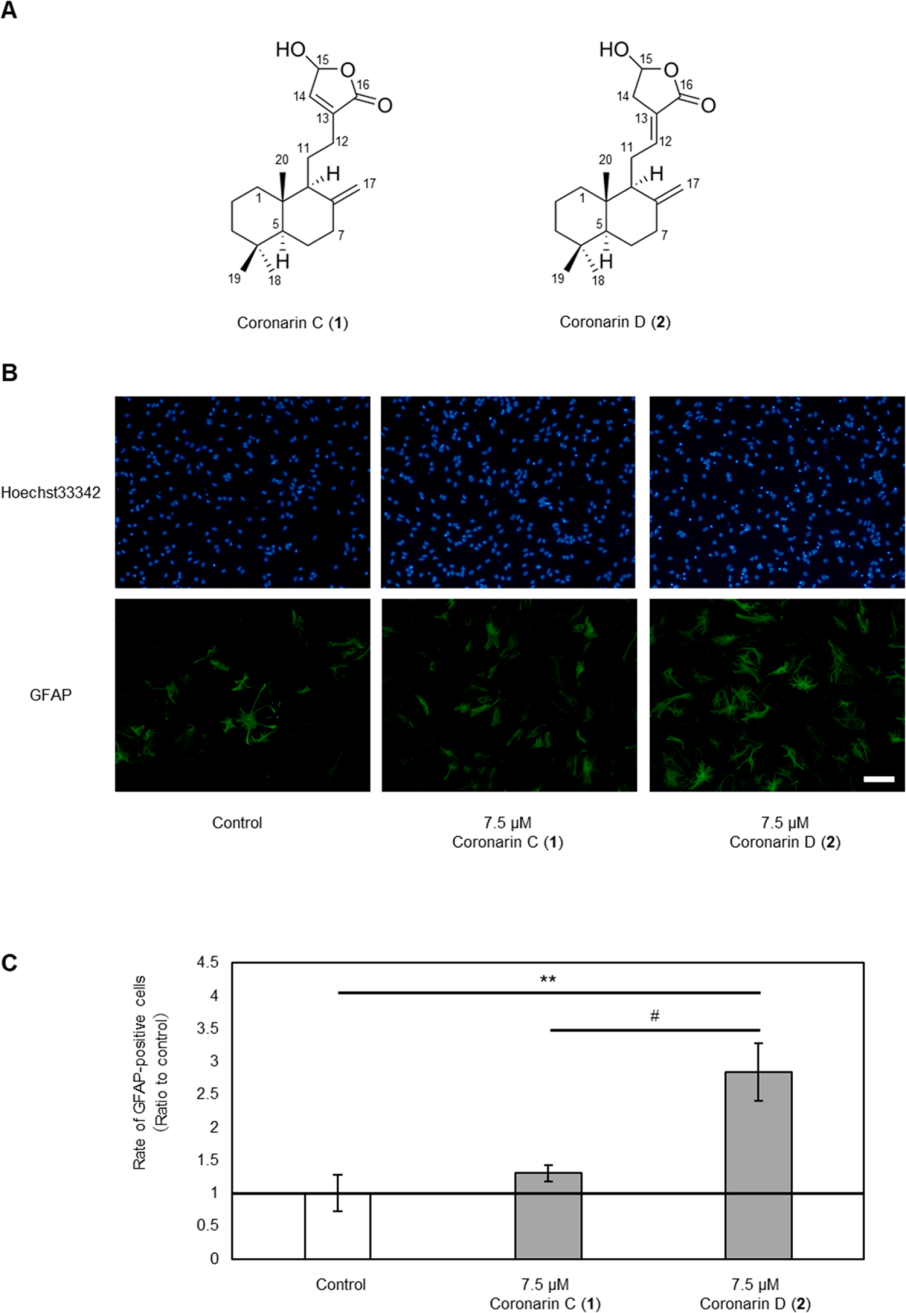
Structures of coronarins C (**1**) and D (**2**) and their effects on cell differentiation. (A) Structures of coronarins C (**1**) and D (**2**). (B) Fluorescent microscopic images of astrocytes differentiated from NSCs. Cells were treated with DMSO (left) or 7.5 μM **1** (middle) or **2** (right) (blue: Hoechst 33342; green: Alexa Fluor 488-labeled GFAP; scale bar: 100 μm). (C) Rates of differentiation into astrocytes. Cells were treated with DMSO, or compounds **1** or **2** at a concentration of 7.5 μM. Ratios of GFAP-positive cells compared with control are shown (*n*  =  3, mean  ±  standard deviation (SD), **p < 0.01 vs control, ^#^*p* < 0.05 vs **1**).

The molecular formula of compound **1** was determined as C_20_H_30_O_3_ by ESI-MS analysis ([M + H]^+^*m*/*z* 319.2265; calculated *m*/*z* 319.2273), and an ion peak of [M – H_2_O + H]^+^ observed at *m*/*z* 301.2164 suggested the presence of hydroxy groups. In the ESI-MS of compound **2**, ion peaks of [M + H]^+^, [M + Na]^+^ and [M – H_2_O + H]^+^ were observed at *m*/*z* 319.2261, 341.2086, and 301.2162, respectively. Thus, compound **2** is an isomer of **1**. The ^1^H NMR spectra showed three singlet methyl signals and two singlet signals for exomethylenes in both **1** and **2**. Combinations of the database search with SciFinder and the analyses of the spectral data identified compounds **1** and **2** as labdane diterpenes, coronarins C, and D, respectively (Figure 2A).^28^ Compound **2** had a greater ability to induce differentiation of NSCs into astrocytes than compound **1** (Figure 2B,C).

### Purification and Identification of Compounds **2** and **3** from the Fresh Rhizomes of the Wild Turmeric *C. aromatica*

To confirm whether compound **2** is naturally occurring, fresh rhizomes of *C. aromatica* (520 g wet weight) were extracted with MeOH. The extract was then subjected to ODS flash chromatography (⌀5.0 cm × 10.0 cm) yielding six fractions (fr.17-1-6). The fourth fraction, fr.17-4, eluted with MeCN/H_2_O (85:15), was further fractionated with silica gel open column chromatography [⌀3.0 cm × 10.0 cm, CHCl_3_, CHCl_3_/MeOH (95:5 and 9:1), CHCl_3_/MeOH/H_2_O (8:2:0.1 and 7:3:0.5), MeOH] to yield 11 fractions (fr.18-1-11). One of the obtained fractions (fr.18-6) was purified by reversed-phase HPLC [MeCN/H_2_O (7:3)] to give fr.19-2, containing **2** as the major component on the basis of liquid chromatography–mass spectrometry (LC–MS) and NMR spectral analyses.

**Figure 3:**
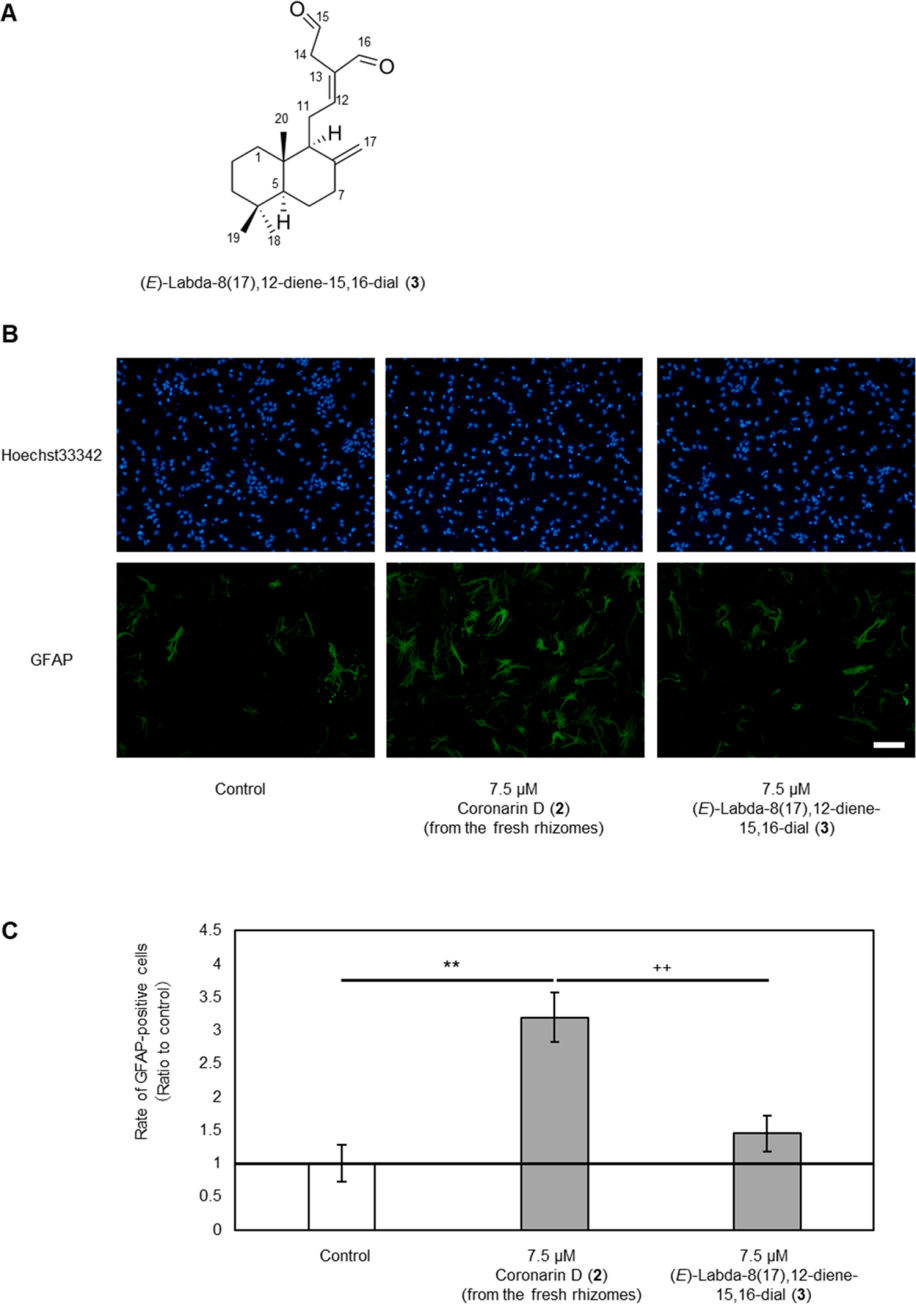
Structure of (*E*)-labda-8(17),12-diene-15,16-dial (**3**) and effects on cell differentiation. (A) Structure of (*E*)-labda-8(17),12-diene-15,16-dial (**3**). (B) Fluorescent microscopic images of astrocytes differentiated from NSCs. Cells were treated with DMSO (left), or 7.5 μM of **2** (from the fresh rhizomes, middle) or **3** (right) (blue: Hoechst 33342; green: Alexa Fluor 488-labeled GFAP; scale bar: 100 μm). (C) Rates of differentiation into astrocytes. Cells were treated with DMSO, or compounds **2** (from the fresh rhizomes) or **3** at a concentration of 7.5 μM. Ratios of GFAP-positive cells compared with control are shown (*n*  =  3, mean  ±  SD, **p < 0.01 vs control, ^++^*p* < 0.01 vs **3**).

Compound **3** was isolated from fr.18-3 by reversed-phase HPLC [MeCN/H_2_O (75:25)] and had a molecular formula of C_20_H_30_O_2_ as determined by ESI-MS analysis (*m*/*z* 303.2322 [M + H]^+^). The ^1^H NMR spectrum of **3** showed the same three singlet methyl signals (δ_H_ 0.87, 0.80, 0.71) and two singlet signals of exomethylenes (δ_H_ 4.85, 4.35) as for **1** and **2**. Inspection of the ^13^C NMR spectrum of **3** indicated the existence of two aldehyde groups (δ_C_ 197.47, 193.74). In addition to this observation, database search with SciFinder identified compound **3** as (*E*)-labda-8(17),12-diene-15,16-dial (Figure 3).^29^ Compound **2** obtained from fresh rhizomes of *C. aromatica* also showed the promoting activity of differentiation of NSCs into astrocytes, but compound **3** had no significant effects (Figure 3B,C).

### Evaluation of Astrocytic Differentiation of NSCs by Compound **2**

**Figure 4:**
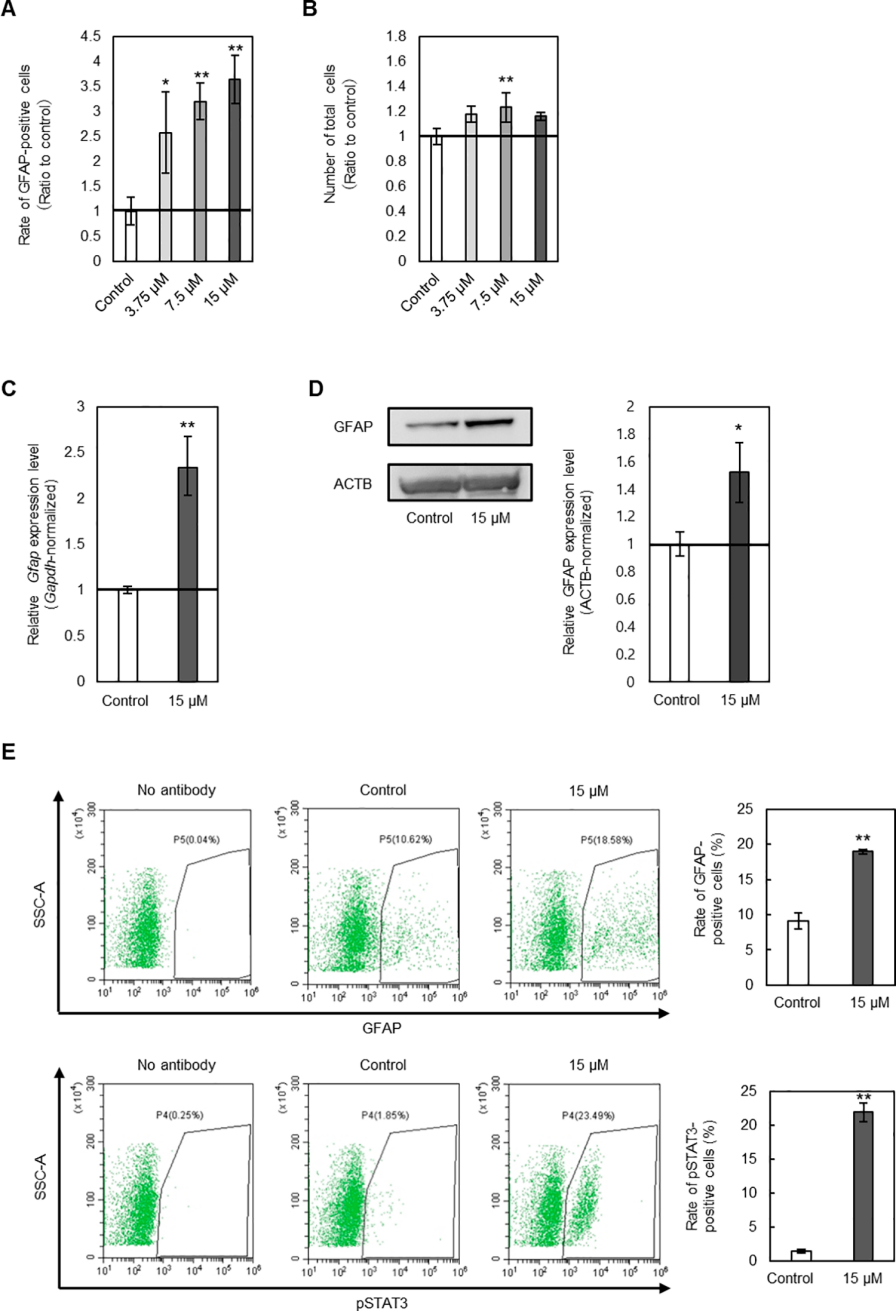
Activities on astrocytic differentiation by compound **2**. (A) Rates of differentiation into astrocytes. Cells were treated with DMSO, or compound **2** at a concentration of 3.75, 7.5, or 15 μM. Ratios of GFAP-positive cells compared with control are shown (*n*  =  3, mean  ± SD, *p < 0.05 vs control, **p < 0.01 vs control). (B) Number of total cells. Cells were treated with DMSO, or **2** at a concentration of 3.75, 7.5, or 15 μM. Number of Hoechst 33342-positive cells compared with control are shown (*n* = 3, mean  ±  SD, **p < 0.01 vs control). (C) Real-time PCR analysis of *Gfap*. Cells were treated with DMSO or 15 μM of **2**. Relative expression levels of *Gfap* normalized to *Gapdh* compared with control are shown (*n*  =  3, mean  ±  SD, **p < 0.01 vs control). (D) Western blotting analysis of GFAP. Cells were treated with DMSO or 15 μM of **2**. Representative bands are shown. Relative expression levels of GFAP normalized to ACTB compared with control are shown (*n*  =  3, mean  ±  SD, *p < 0.05 vs control). (E) Flow cytometry analysis of GFAP and pSTAT3. Cells were treated with DMSO or 15 μM of **2**. Representative plots are shown. Ratios of GFAP or pSTAT3-positive cells are shown (*n*  =  3, mean  ±  SD, **p < 0.01 vs control).

The effects on the differentiation of NSCs were investigated quantitatively for compound **2**. Effects at three concentrations (3.75, 7.5, and 15 μM) of **2** were calculated based on the fluorescence of the microscopic images (Figures 4A and S14). This revealed that compound **2** increased the ratio of GFAP-positive cells in a dose-dependent manner. Since the total number of cells was not decreased by any concentration of compound **2** (Figure 4B), compound **2** was not cytotoxic towards NSCs within the range of the concentrations tested.

To validate the activity of compound **2**, the effects of 15 μM of compound **2** on mRNA and protein expression level of GFAP were examined. Consistent with the result of the *in vitro* NSC differentiation assay, the expression level of GFAP was increased by treatment with compound **2** in mRNA and protein levels (Figure 4C,D). Flow cytometry analysis also revealed that compound **2** treatment enhanced the rates of GFAP-positive cells (Figure 4E). In this analysis, the rate of pSTAT3-positive cells compared to the control condition (1.50 ± 0.259%) was increased (21.9 ± 1.39%, Figure 4E) in the cells treated with compound **2**. It is known that pSTAT3 activates transcription of GFAP.^30^ The elevated level of pSTAT3 caused by compound **2** may play some roles in the promotion of astrocytic differentiation. In contrast, the increases of rates of GFAP or pSTAT3-positive cells by the treatment with compound **2** were not observed in the NSC maintenance medium (Figure S15), which indicates that compound **2** may promote astrocytic differentiation as an assistant in NSCs differentiation medium through JAK/STAT3 signaling.

## Discussion

This is the first report identifying coronarin D (**2**) in the wild turmeric *C. aromatica*, as the bioactive compound promoting the astrocytic differentiation of NSCs. Compound **2** is a labdane diterpene. While many labdane diterpenes have been isolated from various plants in the Zingiberaceae family, such as the Ginger lily *Hedychium coronarium*,^31−35^ they have never been previously reported from *C. aromatica*. Compounds **1** and **2** were previously isolated from the rhizomes of *H. coronarium*(28) and **3** from the seeds of *Alpinia galanga*.^29^ Compound **2** has also been reported from the rhizomes of *Amomum maximum*(36) and *Curcuma amada*.^37^

Biological activities of labdane diterpenes include cytotoxicity against V-79 cells,^28^ anti-inflammatory activity,^38^ inhibition of vascular permeability, NO production,^39^ and inhibition of hexosaminidase release in RBL-2H3 cells.^40^ The reported bioactivity for compound **1** is the inhibition of the proliferation of A-549 cells,^41^ while antibacterial^42,43^ and anti-inflammatory activities^44^ have been reported for compound **2**. Compound **3** shows inhibition against α-glucosidase, lipase,^45^ and the growth of Gram-negative bacteria.^46^

Curcumin^3^ is regarded as the major bioactive compound in *C. aromatica*, but in this study, coronarin D (**2**) was identified as another bioactive substance with a strong ability to promote astrocytic differentiation. Coronarin C (**1**), an isomer of **2**, showed only a weak tendency for promoting the astrocytic differentiation of NSCs. Compounds **1** and **2** differ only in the position of the double bond (Δ^12^ vs Δ^13^), but this difference significantly affects the activity. In addition, (*E*)-labda-8(17),12-diene-15,16-dial (**3**) showed only a weak activity for astrocytic differentiation, suggesting that the 15-hydroxy- Δ^12^-γ-lactone moiety in compound **2** is essential for the bioactivity.

GFAP is a microfilament protein almost in astrocytes in brain tissue, used for the identification of astrocytes *in vivo*, and GFAP-positive astrocytes display a typically stellate morphology.^19^ In the series of experiments, it was found that compound **2** increased the rate of GFAP-positive stellate astrocytes and GFAP expression at the mRNA and protein levels. Flow cytometry analysis showed that compound **2** increased the number of pSTAT3-positive cells. In the JAK-STAT signaling pathway, phosphorylated STAT3 plays the role of the key transcription factor that promotes astrocytic differentiation.^47,48^ This suggests that compound **2** may promote astrocytic differentiation by activating JAK-STAT signaling pathway and phosphorylating STAT3. This pathway is activated when some cytokines (e.g., IL-6 or EGF) bind to receptors such as GP130 or EGFR.^49^ An affinity test of compound **2** with these receptors or a comprehensive expression profiling analysis related to JAK-STAT signaling pathway may help to clarify the mechanism of action for compound **2**.

The relationship between the number of astrocytes in the cerebral cortex and various neurological diseases has garnered increasing attention. Accumulating reports indicate that inflammatory reactions caused by decreased number of normal astrocytes can lead to the development of Alzheimer’s disease,^50^ vulnerability to stress,^51^ and depressive symptoms.^52^ Therefore, bioactive compounds that modulate the astrocytic differentiation of NSCs may have potential in the treatment or prevention of neurodegenerative diseases. For example, AMP-*N*_1_-oxide contained in royal jelly has been reported to promote astrocytic differentiation^53^ and piceatannol found in the seeds of passion fruit promotes the proliferation and differentiation of NSCs into astrocytes.^54^ Coronarin D (**2**) is an additional example of a food component that promotes astrocytic differentiation, and it may be a promising lead compound for the treatment of various neurological diseases or as a supplement for dementia prevention.

## Supporting Information Available

Purification schemes for coronarin C (**1**), coronarin D (**2**), and (*E*)-labda-8(17),12-diene-15,16-dial (**3**) (Figure S1); ESI-MS of coronarin C (**1**) (Figure S2, fr.6-7, positive mode); ^1^H NMR spectrum of coronarin C (**1**) in CDCl_3_ (Figure S3, fr.6-7, 400 MHz); ^13^C NMR spectrum of coronarin C (**1**) in CDCl_3_ (Figure S4, fr.6-7, 600 MHz), ESI-MS of coronarin D (**2**) (Figure S5, fr.6-5, positive mode); ^1^H NMR spectrum of coronarin D (**2**) in CDCl_3_ (Figure S6, fr.6-5, 400 MHz); ^13^C NMR spectrum of coronarin D (**2**) in CDCl_3_ (Figure S7, fr.6-5, 600 MHz); ESI-MS of fr.19-2 (Figure S8, positive mode); ^1^H NMR spectrum of fr.19-2 in CDCl_3_ (Figure S9, 400 MHz); ^13^C NMR spectrum of fr.19-2 in CDCl_3_ (Figure S10, 600 MHz); ESI-MS of (*E*)-labda-8(17),12-diene-15,16-dial (**3**) (Figure S11, fr.20-6, positive mode); ^1^H NMR spectrum of (*E*)-labda-8(17),12-diene-15,16-dial (**3**) in CDCl_3_ (Figure S12, fr.20-6, 400 MHz); ^13^C NMR spectrum of (*E*)-labda-8(17),12-diene-15,16-dial (**3**) in CDCl_3_ (Figure S13, fr.20-6, 600 MHz); fluorescent microscopic images of astrocytes differentiated from NSCs treated with DMSO, or compound **2** at a concentration of 3.75, 7.5, or 15 μM (Figure S14); and ratios of GFAP or pSTAT3-positive cells in the flow cytometry analysis of cells treated with DMSO or 15 μM of **2** in the NSC maintenance medium (*n* =  3, mean  ±  SD, Figure S15) (PDF)

## Abbreviations Used

*C*: *Curcuma*
NSC: neural stem cell
ESC: embryonic stem cell
NMR: nuclear magnetic resonance
MS: mass spectrometry
LC: liquid chromatography
ESI: electrospray ionization
MeOH: methanol
H_2_O: water
MeCN: acetonitrile
CHCl_3_: chloroform
ODS: octadecylsilyl
TLC: thin-layer chromatography
HPLC: high-performance liquid chromatography
DMEM: Dulbecco’s modified Eagle’s medium
FBS: fetal bovine serum
P/S: penicillin/streptomycin
LIF: leukemia inhibitory factor
EB: embryoid body
rhEGF: recombinant human epidermal growth factor
rhFGF-2: recombinant human fibroblast growth factor-2
DMSO: dimethyl sulfoxide
PBS: phosphate-buffered saline
GFAP: glial fibrillary acidic protein
PCR: polymerase chain reaction
RNA: ribonucleic acid
cDNA: complementary deoxyribonucleic acid
SDS-PAGE: sodium dodecyl sulfate-polyacrylamide gel electrophoresis
PVDF: poly(vinylidene fluoride)
HRP: horseradish peroxidase
