# Supplementary material for: Coronarin D, a Metabolite from the Wild Turmeric, Curcuma aromatica, Promotes the Differentiation of Neural Stem Cells into Astrocytes
Source: J Agric Food Chem. 2022 Mar 4;70(10):3300–9. doi: 10.1021/acs.jafc.2c00020 (PMC8931754; doi:10.1021/acs.jafc.2c00020)
Supplement: Supplementary file 1 — jf2c00020_si_001.pdf [file jf2c00020_si_001.pdf]

Supporting Information

Coronarin D, a metabolite from the wild turmeric, *Curcuma aromatica*, promotes the differentiation of neural stem cells into astrocytes

Satoshi Otsuka, Midori Kawamura, Shutaro Fujino, Fumiaki Nakamura, Daisuke Arai, Nobuhiro Fusetani, and Yoichi Nakao*

Contents

Figure S1. Purification schemes for coronarin C (**1**), coronarin D (**2**), and (*E*)-labda-8(17),12-diene-15,16-dial (**3**).

Figure S2. ESI-MS of coronarin C (**1**) (fr.6-7, positive mode).

Figure S3. ^{1}H NMR spectrum of coronarin C (**1**) in $CDCl_3$ (fr.6-7, 400 MHz).

Figure S4. ^{13}C NMR spectrum of coronarin C (**1**) in $CDCl_3$ (fr.6-7, 600 MHz).

Figure S5. ESI-MS of coronarin D (**2**) (fr.6-5, positive mode).

Figure S6. ^{1}H NMR spectrum of coronarin D (**2**) in $CDCl_3$ (fr.6-5, 400 MHz).

Figure S7. ^{13}C NMR spectrum of coronarin D (**2**) in $CDCl_3$ (fr.6-5, 600 MHz).

Figure S8. ESI-MS of fr.19-2 (positive mode).

Figure S9. ^{1}H NMR spectrum of fr.19-2 in $CDCl_3$ (400 MHz).

Figure S10. ^{13}C NMR spectrum of fr.19-2 in $CDCl_3$ (fr.19-2, 600 MHz).

Figure S11. ESI-MS of (*E*)-labda-8(17),12-diene-15,16-dial (**3**) (fr.20-6, positive mode).

Figure S12. ^{1}H NMR spectrum of (*E*)-labda-8(17),12-diene-15,16-dial (**3**) in $CDCl_3$ (fr.20-6, 400 MHz).

Figure S13. ^{13}C NMR spectrum of (*E*)-labda-8(17),12-diene-15,16-dial (**3**) in $CDCl_3$ (fr.20-6, 600 MHz).

Figure S14. Fluorescent microscopic images of astrocytes differentiated from NSCs treated with DMSO, or compound **2** at a concentration of 3.75, 7.5, or 15 μM.

Figure S15. Ratios of GFAP or pSTAT3-positive cells in the flow cytometry analysis of cells treated with DMSO or 15 μM of **2** in the NSC maintenance medium (n = 3, mean ± S.D.).

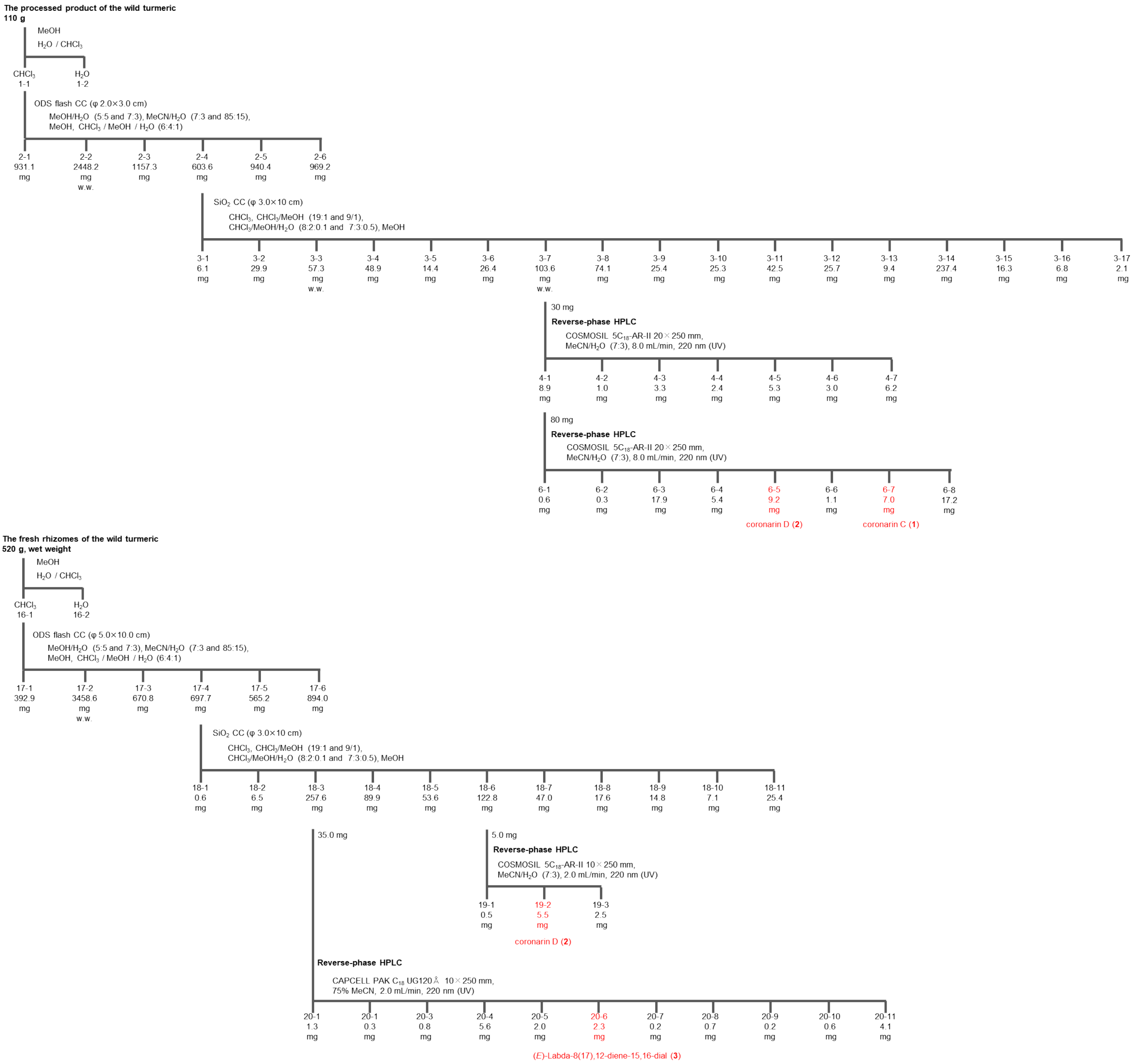

Figure S1. Purification schemes for coronarin C (**1**), coronarin D (**2**), and (*E*)-labda-8(17),12-diene-15,16-dial (**3**).

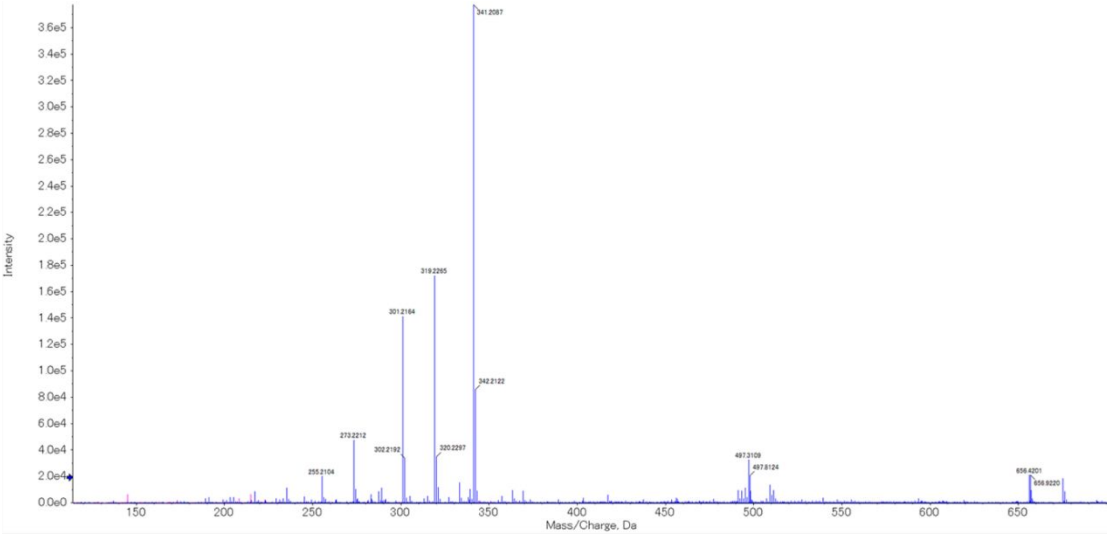

Figure S2. ESI-MS of coronarin C (**1**) (fr.6-7, positive mode).

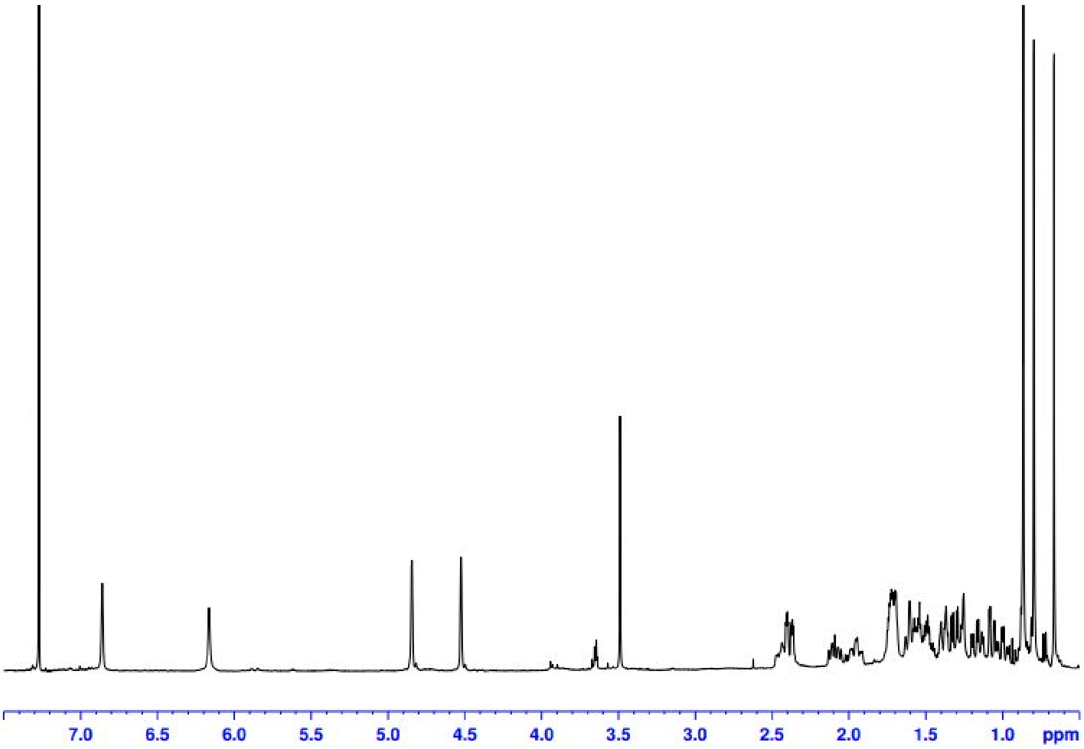

Figure S3. ^{1}H NMR spectrum of coronarin C (**1**) in $CDCl_3$ (fr.6-7, 400 MHz).

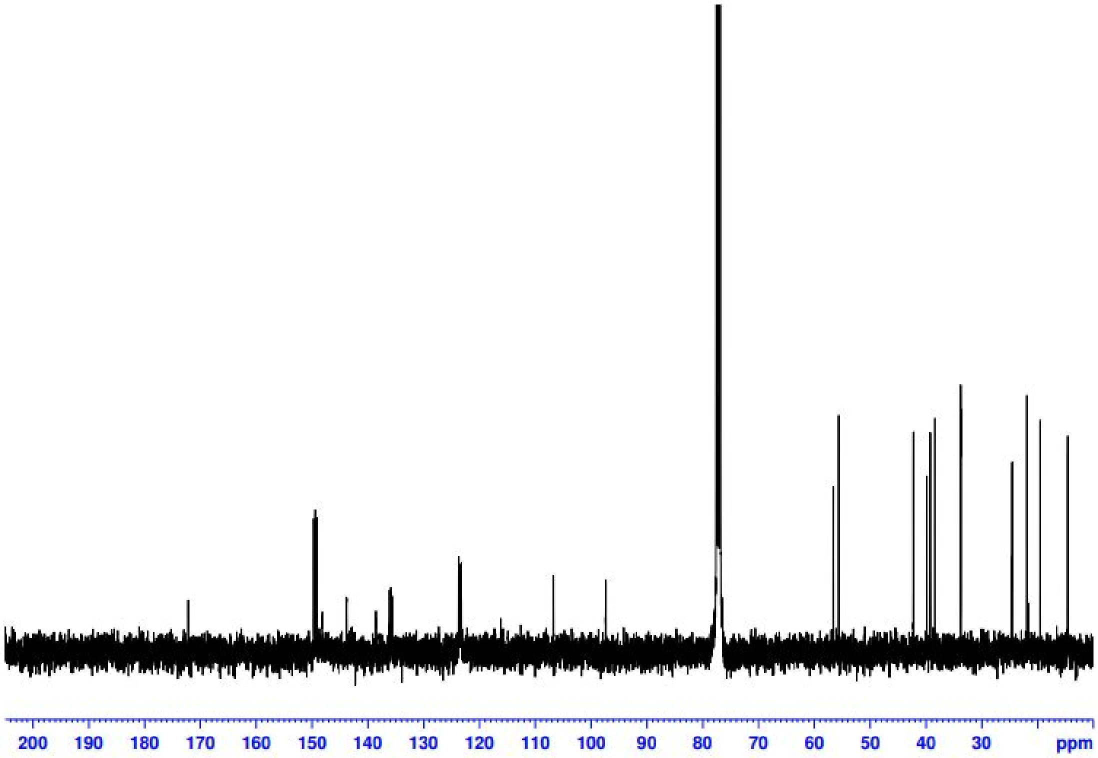

Figure S4. 13C NMR spectrum of coronarin C (**1**) in $CDCl_3$ (fr.6-7, 600 MHz).

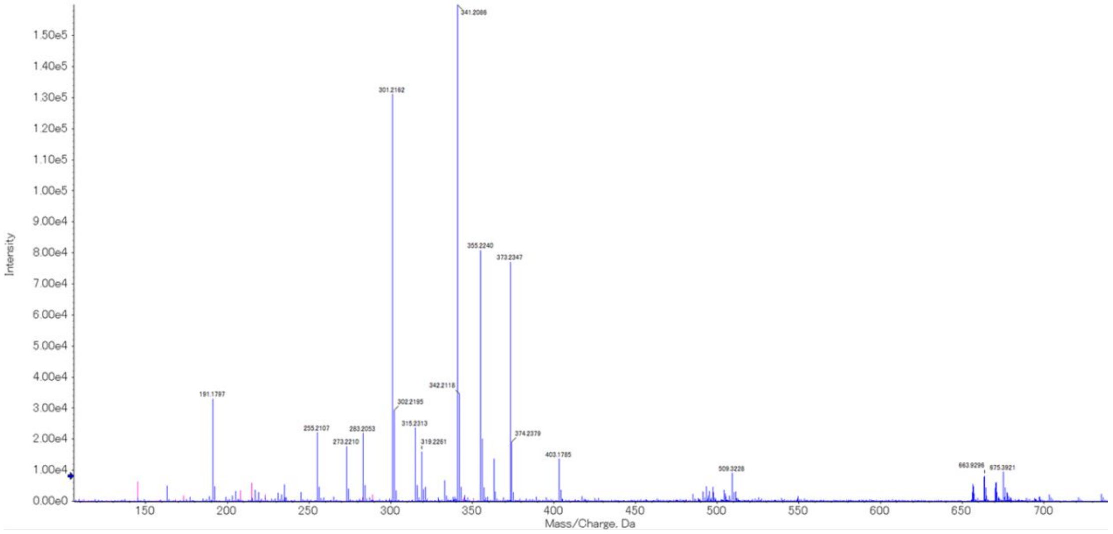

Figure S5. ESI-MS of coronarin D (**2**) (fr.6-5, positive mode).

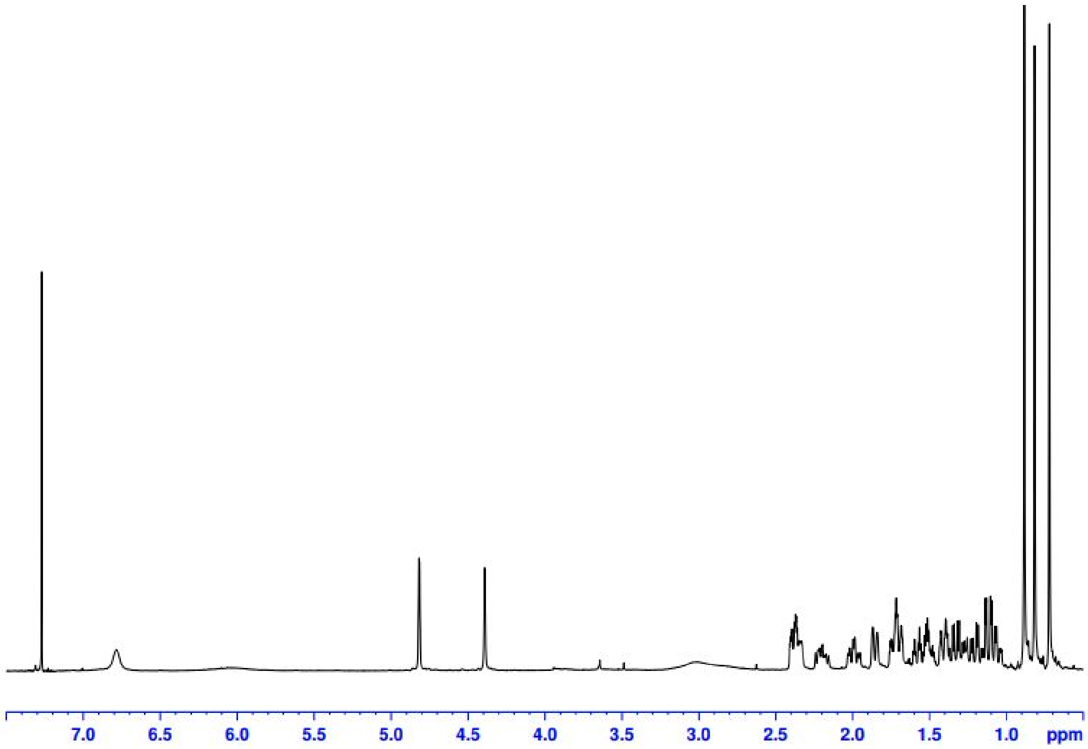

Figure S6. ^{1}H NMR spectrum of coronarin D (**2**) in $CDCl_3$ (fr.6-5, 400 MHz).

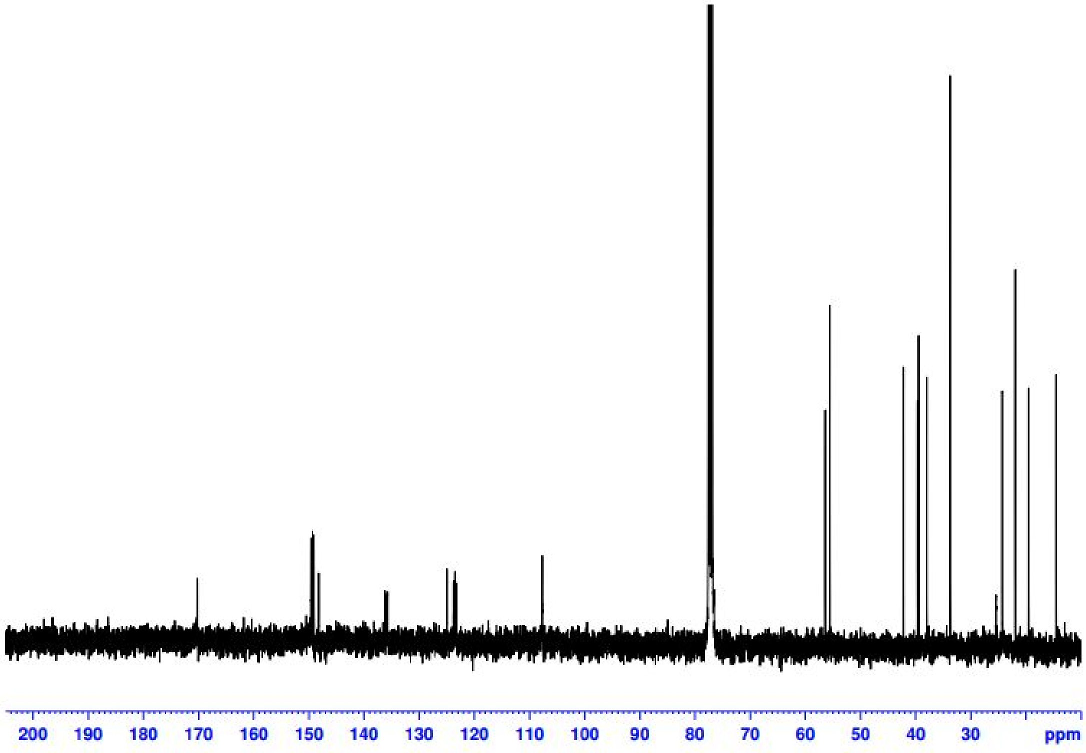

Figure S7. ^{13}C NMR spectrum of coronarin D (**2**) in $CDCl_3$ (fr.6-5, 600 MHz).

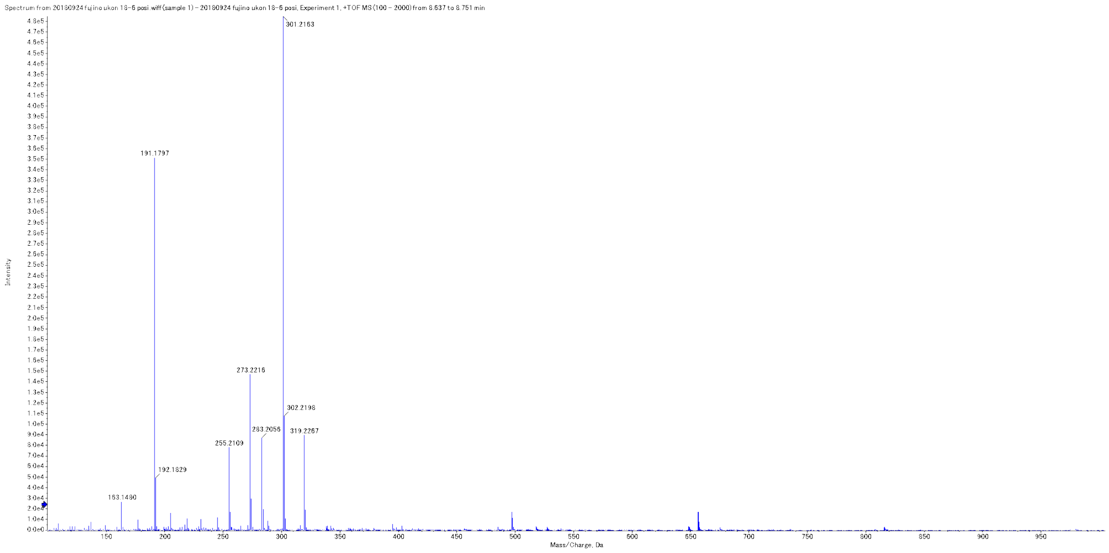

Figure S8. ESI-MS of fr.19-2 (positive mode).

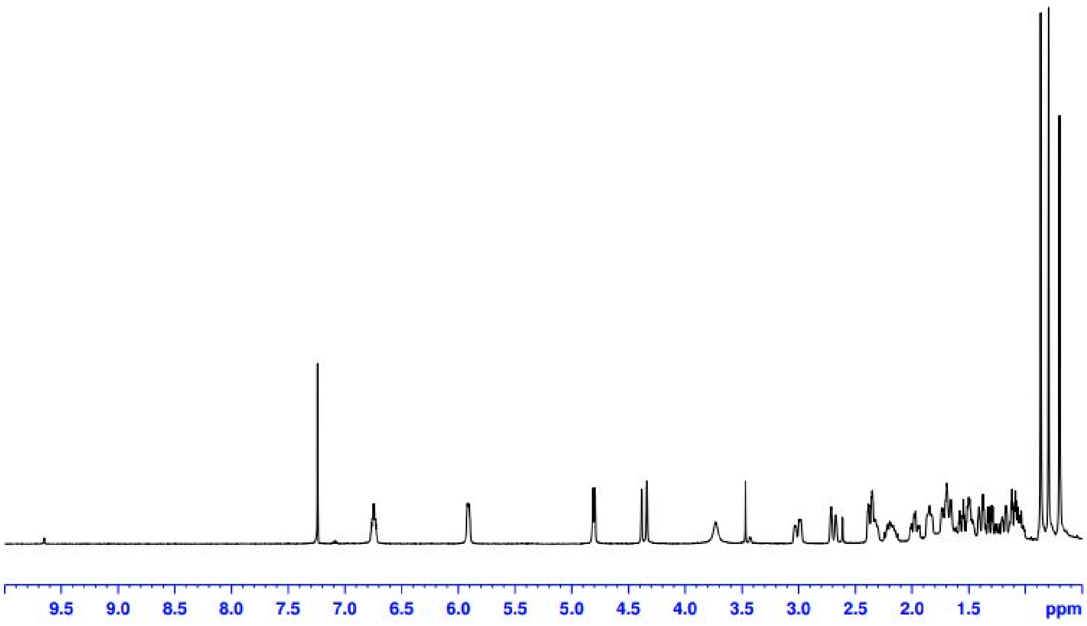

Figure S9. ^{1}H NMR spectrum of fr.19-2 in $CDCl_3$ (400 MHz).

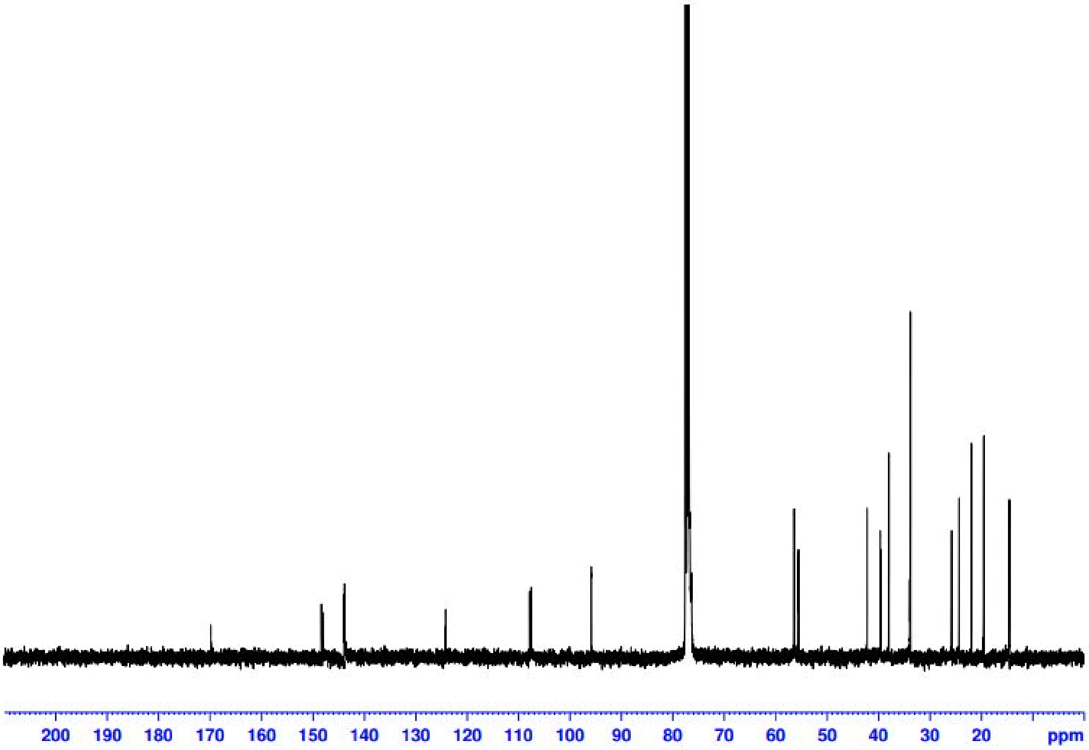

Figure S10. ^{13}C NMR spectrum of fr.19-2 in $CDCl_3$ (600 MHz).

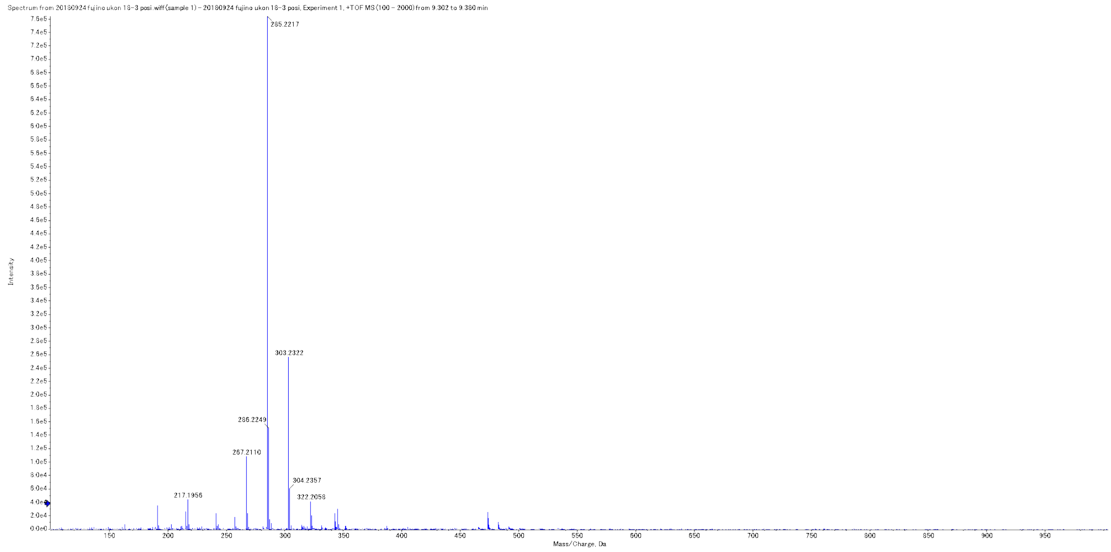

Figure S11. ESI-MS of (*E*)-labda-8(17),12-diene-15,16-dial (**3**) (fr.20-6, positive mode).

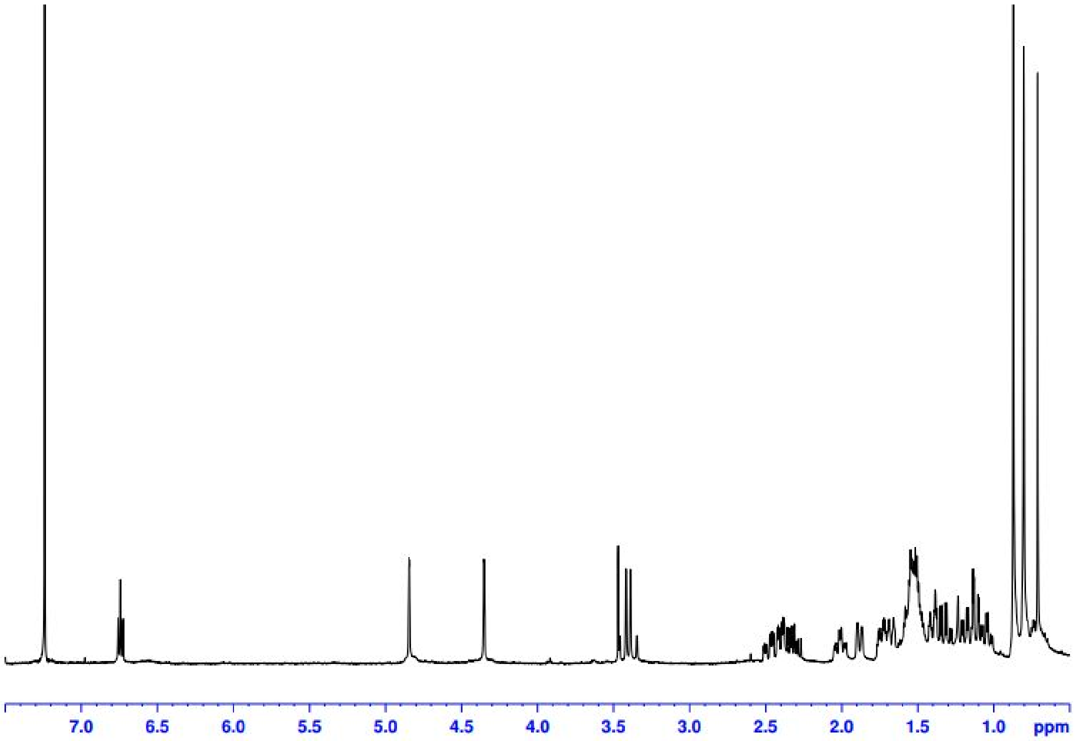

Figure S12. ^{1}H NMR spectrum of (*E*)-labda-8(17),12-diene-15,16-dial (**3**) in $CDCl_3$ (fr.20-6, 400 MHz).

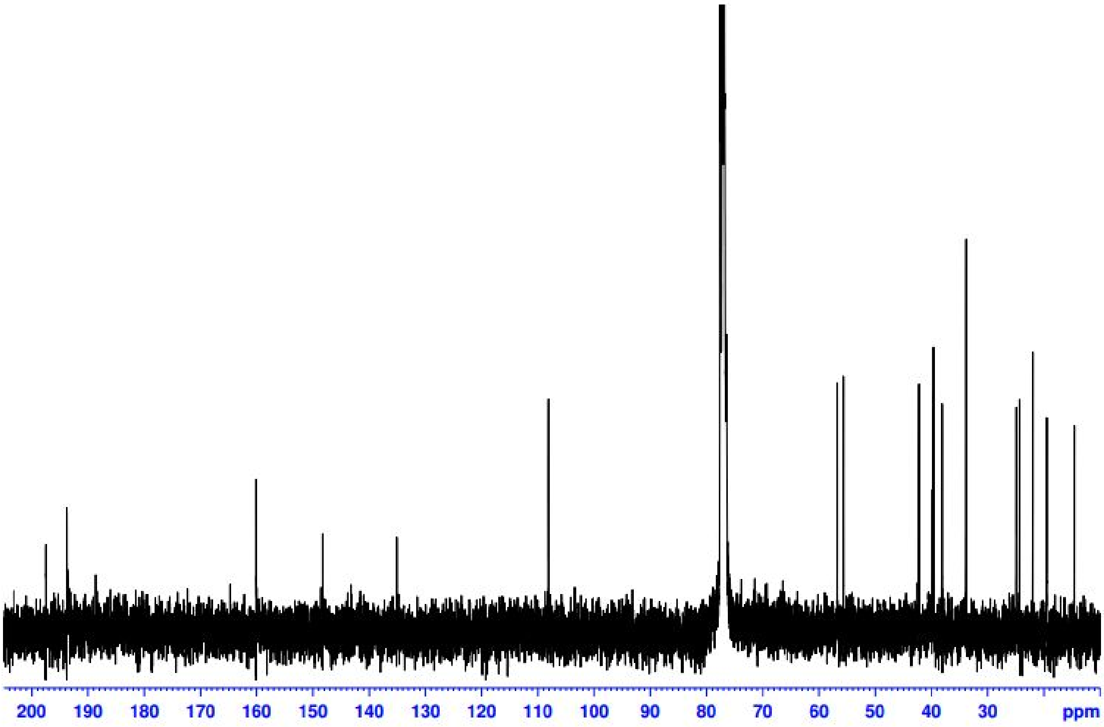

Figure S13. ^{13}C NMR spectrum of (*E*)-labda-8(17),12-diene-15,16-dial (**3**) in $CDCl_3$ (fr.20-6, 600 MHz).

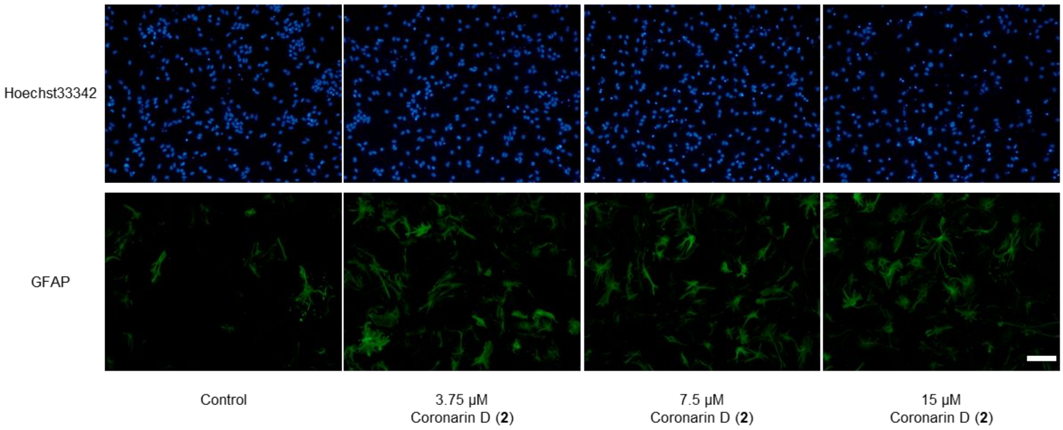

Figure S14. Fluorescent microscopic images of astrocytes differentiated from NSCs treated with DMSO, or compound **2** at a concentration of 3.75, 7.5, or 15 μM.

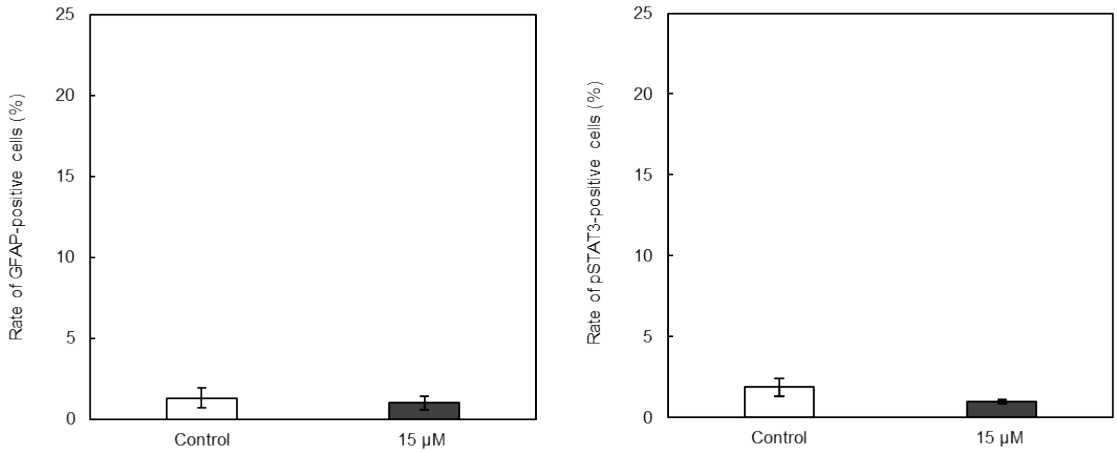

Figure S15. Ratios of GFAP or pSTAT3-positive cells in the flow cytometry analysis of cells treated with DMSO or 15 μM of **2** in the NSC maintenance medium (n = 3, mean ± S.D.).
